# How Does Exposure to Dementia Relate to Subjective Cognition? A Systematic Review

**DOI:** 10.1093/geroni/igad056

**Published:** 2023-06-19

**Authors:** Jennifer R Turner, Nikki L Hill, Leslie Brautigam, Sakshi Bhargava, Jacqueline Mogle

**Affiliations:** Edna Bennett Pierce Prevention Research Center, College of Health and Human Development, Pennsylvania State University, University Park, Pennsylvania, USA; Department of Psychology, College of Arts and Sciences, University of Hawaiʻi at Hilo, Hilo, Hawaii, USA; Ross and Carol Nese College of Nursing, Pennsylvania State University, University Park, Pennsylvania, USA; Ross and Carol Nese College of Nursing, Pennsylvania State University, University Park, Pennsylvania, USA; Department of Patient-Centered Outcomes Assessment, RTI Health Solutions, Research Triangle Park, North Carolina, USA; Edna Bennett Pierce Prevention Research Center, College of Health and Human Development, Pennsylvania State University, University Park, Pennsylvania, USA; Department of Psychology, College of Behavioral, Social, and Health Sciences, Clemson University, Clemson, South Carolina, USA

**Keywords:** Alzheimer’s disease, Cognition, Dementia, Family history, Subjective cognitive decline

## Abstract

**Background and Objectives:**

Subjective cognitive decline (SCD) may be indicative of future objective cognitive decline. However, factors other than objective cognitive performance may influence SCD. This review addresses whether family history or close, nonfamilial exposure to dementia is associated with self-reported SCD.

**Research Design and Methods:**

Searches were conducted in PubMed, PsycINFO, Web of Science, and the Dissertations and Theses database. Eligible articles included measures of self-reported cognition for community-dwelling middle-aged or older adults (40+ years) not diagnosed with dementia, and who had either a family history of dementia, a family member, spouse, or close friend with dementia. The quality of evidence was evaluated using the LEGEND Appraisal Tool. Evidence was synthesized narratively.

**Results:**

A total of 32 articles were included, with 28 rated as *good quality*. Across studies, the relationship between dementia exposure and SCD was inconsistent. A significant association between exposure and SCD was found in 6 studies; however, 17 reviewed studies found no evidence of a relationship. The remaining 9 studies found mixed associations. Modifying factors that could potentially influence these associations were exploratorily identified among studies to provide context to our results. These factors included dementia worry, emotional closeness, and measurement sensitivity.

**Discussion and Implications:**

Findings of this review suggest that both first-degree relatives and spouses of persons with dementia may have an increased likelihood of reporting SCD, although the current heterogeneity in definitions of exposure to dementia and SCD may influence these findings. In addition to the relationship between dementia exposure and SCD, future research should examine potential modifiers, including meaning attributed to exposure, as identifying how these perceptions affect cognition may promote early intervention.

**Translational Significance:** Subjective cognitive decline (SCD) is a self-perceived worsening of cognition and is associated with an increased risk of dementia. Distinguishing factors that influence SCD reporting may aid the identification of individuals potentially at risk for poorer cognitive and functional outcomes. This review investigates how exposure to individuals with dementia, through family history or proximity, may relate to SCD and also describes several potential modifiers of this relationship. A better understanding of the factors that affect SCD reporting may contribute to a more precise, yet holistic, measurement of preclinical changes in cognition and help inform best practices for cognitive screening and help-seeking in clinical settings.

Many people are affected by mild cognitive impairment (MCI) or dementia, including Alzheimer’s disease (AD), in later life. At the same time, an increasingly larger share of the population is *exposed* to dementia, either through family history or physical and social proximity to someone who has cognitive impairment. *Dementia exposure* is a broad term that can be conceptualized as either familial (i.e., first-degree relatives such as parent, sibling, or child) or nonfamilial (i.e., spouse, partner, and close friend) familiarity with someone with dementia, but can also encapsulate emotional closeness and the frequency of contact (such as a caregiver). Some research has suggested that close familial proximity to someone with dementia, such as a parent, can spark fear of developing AD in later life (Kinzer & Suhr, 2016) or heighten one’s awareness of possible signs of dementia in themselves (Hodgson & Cutler, 2003), yet the examination of whether dementia exposure is associated with current perceptions of cognitive performance or decline has not been comprehensively reviewed.

Subjective cognition is most often examined and defined as subjective cognitive decline (SCD) or subjective cognitive impairment (SCI; Jessen et al., 2014). Older adults reporting declines in their cognition are 2–4 times more likely to develop objective cognitive impairments such as MCI or dementia (Jessen et al., 2020). This suggests that early perceptions of cognitive decline may represent a prodromal stage of objective cognitive decline for some older adults that can last from 2 to 20 years (Reisberg & Gauthier, 2008). Growing knowledge has led to the refinement of the study of SCD, including the development of the SCD-*plus* criteria that consider the type of cognitive complaints and presence of the apolipoprotein E (APOE) e4 genotype, for example, to improve SCD as a predictor of AD (Jessen et al., 2014). Complementary to SCD-related factors that increase its predictive utility is our need to understand factors that confound interpretation.

SCD is an early indicator of cognitive decline and dementia in some individuals, but it is highly heterogeneous in terms of the underlying causes (Jessen et al., 2014). People may report SCD for many reasons, including affective disorders (e.g., depression; Hill et al., 2016) or behavioral tendencies associated with certain personality traits (e.g., neuroticism; Koller et al., 2019). Similarly, dementia exposure can influence one’s personal experience with cognitive performance. For example, older adults with a first-degree relative with dementia maybe more likely to report memory decline, even when they have no objective cognitive deficits (Hill et al., 2016). This may be related to perceived familial risk because having a spouse with AD may not have a similar impact on SCD reporting (Tsai et al., 2006). However, the evidence regarding dementia exposure and SCD has not been synthesized to understand potentially conflicting evidence or determine the next steps.

Together, given that SCI and SCD may be indicative of a prodromal stage of dementia or AD (Jessen et al., 2014, 2020), it is important to examine pathways that may affect perceptions of cognition. Identifying these features can promote early identification of those most likely to be affected and provide targets for more specifically tailored interventions. Individuals with dementia exposure, either genetically or through social proximity and interaction, may be one such population. Thus, the primary aim of the present systematic review was to evaluate and synthesize the current evidence regarding the relationship between dementia exposure and self-reported cognition (hereafter referred to as SCD) among adults without evidence of objective cognitive impairment. A secondary, exploratory aim was to identify factors that could potentially buffer or strengthen this relationship, such as the type of relationship (e.g., familial vs nonfamilial exposure) or length and proximity to the person with dementia. See Figure 1 for a graphical illustration and conceptual model of these potential relationships.

**Figure 1.**
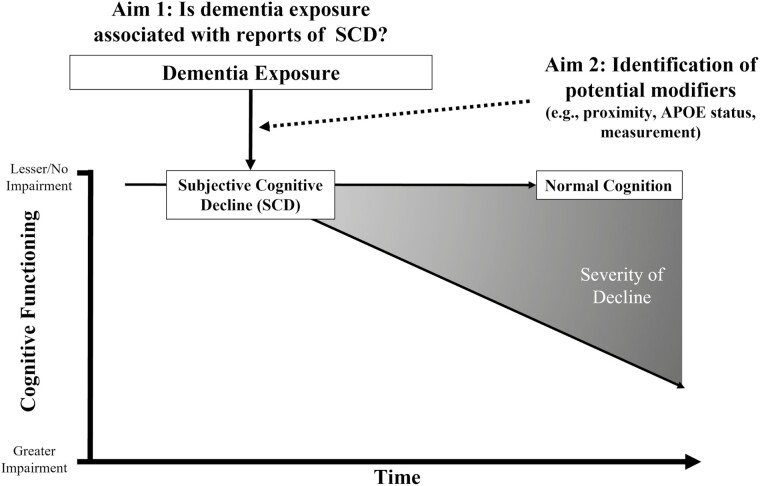
Conceptual model of the relationship between dementia exposure and subjective cognitive decline (SCD). Arrows in model represent hypothetical trajectories of cognition for individuals with and without dementia exposure (e.g., individuals with SCD may maintain normal cognition or progress to objective cognitive impairment). The dashed line for Aim 2 indicates that this aim was exploratory and based on observed commonalities within included manuscripts (i.e., *data-driven*). Gradient represents hypothetical range of cognitive decline based on SCD status and potential modifiers of dementia exposure.

## Method

PRISMA guidelines, updated for 2020 (Page et al., 2021), were followed in order to conduct this systematic review (see Supplementary Material for the PRISMA checklist). Prior to conducting the literature search, the review protocol was registered with PROSPERO (ID: CRD42021232329).

### Search Strategy

Searches were originally conducted in MEDLINE (PubMed), PsycINFO (ProQuest), Web of Science, and the Dissertations and Theses Database on March 5–6, 2021. Updated searches were conducted on January 26, 2023 within the same databases. Results were limited to studies of middle-aged and older adults (40+ years) and published in the English language. The following search strategy was used: ([cognit* OR memory OR dementia OR Alzheimer*] AND [subjective OR complaint*] AND [family OR caregiv* OR community OR expos*]) AND la.exact(“English”) AND age.exact(“Aged [65 Yrs & Older]” OR “Very Old [85 Yrs & Older]” OR “Middle Age [40–64 Yrs]”). In addition, we reviewed reference lists of included studies, as well as previous review articles relevant to our review purpose, for additional potential studies for inclusion.

### Selection Criteria

For this review, selected studies met the following inclusion criteria: (1) sample of community-dwelling middle-aged or older adults (40+ years), (2) measure of self-reported cognition, (3) measure of dementia exposure; and (4) reported on the association of self-reported cognition with dementia exposure. Studies were excluded if participants had a diagnosis of cognitive impairment. For this review, studies could be cross-sectional or longitudinal, observational, qualitative, quantitative, or mixed methods. Systematic reviews, meta-analyses, clinical trials and interventions, protocols and editorials, and case studies were not eligible for inclusion.

### Selection and Data Extraction

Selection and evaluation of studies were performed in Covidence software (Covidence, n.d.). Duplicate studies were identified and excluded, and studies that used the same sample within multiple manuscripts were also excluded. First, two authors independently reviewed the titles and abstracts of studies to determine eligibility. Next, the authors reviewed the full text of the articles to determine eligibility. Any disagreements were resolved by a third author.

The following data were initially extracted from the included studies: author and year of publication, type of sample (e.g., community-based), location (i.e., country), sample size, mean age and standard deviation (as available), type of dementia exposure (e.g., family history vs close friend), SCD measures, the relationship between SCD and dementia exposure (e.g., correlations between variables, mean differences, and regression estimates), and general study conclusions and limitations. To provide context to our results, we secondarily extracted data that pertained to potential modifying factors of interest (i.e., variables that may influence the association between dementia exposure and SCD). These variables included: (1) whether the SCD measure was single versus multi-item, (2) primarily targeted memory or cognition more generally, (3) if *dementia worry* was included, (4) whether dementia exposure was conceptualized as genetic versus nongenetic (including if APOE status was assessed) or if proximity was assessed, and (5) if the exposure “type” specified AD. This secondary data extraction was conducted exploratorily. Data were extracted by two authors, and disagreements were resolved by a third author.

### Quality Appraisal

The *Let Evidence Guide Every New Decision* (LEGEND) Appraisal Tool was used to evaluate the quality of evidence in each study (Clark et al., 2009). The LEGEND quality of evidence criteria focuses on validity, reliability, and applicability factors, such as clearly defined methods, objective, unbiased measurement, sufficient sample size and characteristics, appropriate analysis methods, and statistical as well as clinical significance. The tool is primarily used to support clinical decision-making, yet its flexibility in use for synthesizing and comparing across many different research designs (e.g., case-control, cross-sectional, and longitudinal) underpins our selection of this tool for the present usage. Specifically, we used the following components of the LEGEND tool: *Overview* (i.e., relevance of the study to the question of interest), *Reliability*, and *Validity* (see Supplementary Table 1 for a summary of appraisal categories and items). Quality appraisal was conducted in the same manner as data extraction: two authors appraised the article, and disagreements were resolved by a third author. Interrater agreement was 84.4% (agreement on the quality of 27 out of 32 appraisals).

### Narrative Synthesis

Using the data extracted from the Covidence software, and following our conceptual model (Figure 1), a narrative synthesis of results was used to integrate findings across studies. First, we reviewed and tabulated the studies that found a significant relationship between dementia exposure and SCD, a null or nonsignificant relationship between dementia exposure and SCD, or an unclear relationship between dementia exposure and SCD (i.e., evidence of both positive and negative effects). Following the initial extraction, other categories that were tabulated for this synthesis included the number of studies utilizing a familial definition of exposure (and proximity of the relationship: 1st degree, 2nd degree, family history-unspecified) compared with the number of studies using a nonfamilial definition. Next, we divided the type of SCD measure into those that assessed memory compared with cognition, and whether the measure was assessed using a single or multi-item scale. Third, we computed the number of studies that assessed APOE status, whether AD was the specified form of dementia, and whether dementia worry was assessed. Finally, we drew conclusions regarding the pattern of results, including similarities and differences, based on the strength of the evidence for each category based on the synthesis of the results and quality appraisal.

## Results

### Overview of Reviewed Studies

**Figure 2.**
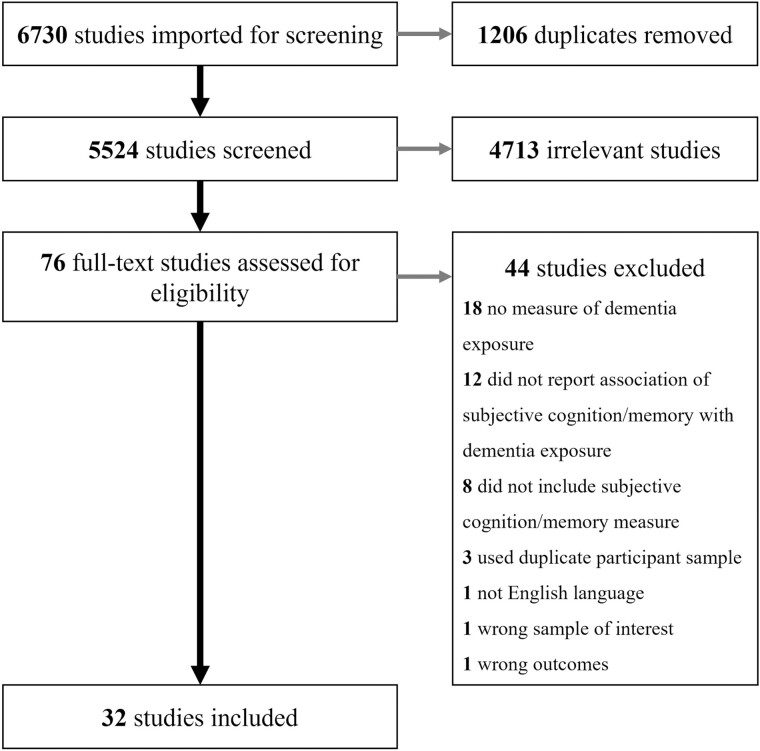
PRISMA flowchart for the current study.

A total of 6,730 studies were initially identified, of which 1,206 were duplicates, and 5,448 were deemed irrelevant to the present review during the title and abstract screening stage. A total of 76 studies were identified for full-text review and the final review consisted of 32 studies that met the inclusion criteria and were extracted for this review (see Figure 2 for PRISMA flowchart). All included studies were quantitative, and most studies were cross-sectional (*n* = 25); studies are identified by their corresponding number in Tables 1 and Supplementary Table 2 based on the observed relationship between dementia exposure and SCD.

**Table 1.** Reviewed Articles with Significant Relationships Between Dementia Exposure and SCD

Reference	Sample type, location	Sample size; age (*SD*)	Study design	Dementia exposure	SCD measures (# items)	Quality appraisal	Analyses and general conclusions
Section 1: Exclusive familial exposure (Articles 1–4)							
1. Bell et al. (2022)	Community (United States)	*n* = 3,809; 66.1 (1.9)	Longitudinal	First degree	How would you rate your memory at the present time? Compared with (previous wave/two years ago), would you say your memory is better now, about the same, or worse than it was then?	Good quality	Family history of dementia was associated with greater perceived memory decline (OR = 1.21, 95% CI: 1.03, 1.42, *p* ≤ .05).
2. Bharambe and Larner (2018)	Clinic (United Kingdom)	*n* = 89; 71.3 (8.1)	Cross-sectional	Family history	In general, how would you rate your memory?	Good quality	Using χ^2^ analyses^a^, participants with functional cognitive disorder were more likely to be classified with SMC (*p* < .01) and have dementia exposure (*p* < .001).
3. Freitas et al. (2012),	Community (Portugal)	*n* = 650; 55.8 (15.1)	Cross-sectional	First degree	SMC scale (10-items)	Good quality	Family history correlated with SMC participant scores (*r* = 0.127, *p* < .01), but not SMC informant scores (*r* = 0.095, *p* = . 24).
4. Haussmann et al. (2018)	Clinic and community (Germany)	aMCI: *n* = 35; 70.3 (6.5) CN: *n* = 40; 66.2 (7.5)	Cross-sectional	First degree	Do you experience subjective memory impairment?	Good quality	There was no difference in family history between CN and aMCI (*p* = .54)^a^. An interaction between group and family history in SMI (*F* = 4.6, *p* = .035) was found: CN without exposure had less severity of SMI (*p* < .001), while CN with family history did not differ from rates found in aMCI (*p* = .85).
Section 2: Familial and nonfamilial exposure (Articles 5 and 6)							
5. Ostergren et al. (2017)	Community (United States)	*n* = 1,641; 64.4 (0.4)	Cross-sectional	First degree; spouse or someone they know	How would you rate your memory at the present time?	Good quality	SMC was associated with perceived risk of AD (*r* = 0.127, *p* < .01). This association was moderated by exposure. Perceived risk was greater among those with spousal or 1^st^ degree exposure (*b* = 0.27, 95% CI: 10.45); knowing someone did not differ from no exposure (*b* = 0.11, 95% CI: −0.01, 0.26).
6. Vitaliano et al. (2017)	Community (United States)	Caregiver: *n* = 122; 71.7 (8.9) Noncaregiver: *n* = 117; 70.2 (7.2)	Longitudinal; subjective cognition measured once.	Spouse	“difficulties (‘*yes*–*no*’) in concentration, attention, forgetting, disorientation, not completing things, reacting slowly, confusion, and making mistakes” (p. 640).	Good quality	Using mean difference comparisons (i.e., ANCOVA), CGs reported greater problems with subjective cognition, *F*(1, 224) = 6.61, *p* < .01, η^2^ = 0.03.

*Notes*: AD = Alzheimer’s disease; aMCI = amnestic mild cognitive impairment; ANCOVA = analysis of covariance; CN = cognitively normal; SCD = subjective cognitive decline; SIME = short inventory of memory experiences; SMC = subjective memory complaints; SMI = subjective memory impairment; Quality appraisal determined using the LEGEND framework; first degree = parent, child, and sibling; second degree = aunt, uncle, grandparent, niece, and nephew.

^a^Indicates that only significance values, not specific results and estimates (e.g., χ^2^, *t*-test, *F-*value, etc.), were presented.

The sample size of the reviewed articles ranged from 30 to 93,604; the majority of studies recruited participants from community-based settings (*n* = 23), some recruited from clinic-based settings (*n* = 4), others recruited from both clinic- and community-based settings (*n* = 5), but all participants were community-dwelling and nonhospitalized. Overall, the included studies were conducted in 10 countries: United States (*n* = 18), Germany (*n* = 4), China (*n* = 2), Spain (*n* = 1), United Kingdom (*n* = 1), Portugal (*n* = 1), the Netherlands (*n* = 1), Australia (*n* = 1), Canada (*n* = 1), and Israel (*n* = 1). One study was conducted in the United States, Germany, and Canada.

Most studies measured participants’ exposure to dementia in a first-degree relative (*n* = 15). In addition, some studies also measured exposure to dementia in a second-degree relative, or a close relative, or a close friend or spouse (*n* = 8). Some studies did not specify the proximity of the relationship with the person with dementia (*n* = 8). One study examined dementia exposure in spouses only (Study 6). SCD was measured using multi-item questionnaires in most studies (*n* = 22) that ranged from 3 to 64 items. Other studies used single-item measures to examine SCD.

### Results of Quality Appraisal

Of the 32 studies reviewed, 87.5% (*n* = 28) were rated as being *good quality* per the adapted LEGEND guidelines (i.e., a score of 9 or higher out of 15 possible; Supplementary Table 1), while only four were considered to be of *lesser quality* because of unclear methodology, small sample size, or other potential threats to validity and reliability. As the determination of good versus lesser quality is based on the consideration of each “no” or “unknown” response compared with the number of affirmative “yes” responses, the sub-categories (i.e., overview, validity, and reliability) were also evaluated. Regarding the *overview* (i.e., relevance) category, only one study (Study 26) earned a rating of less than two out of three possible points. For the *validity* ratings, 56.3% of the studies (*n* = 18) earned a score of five or six (out of 7 possible), while 34.4% (*n* = 11) received the maximum score, indicating high confidence in the studies’ methodology and instruments of assessment. Finally, *Reliability* ratings (which encompassed statistical analyses and results) were moderately high, *n* = 25 (78.1%) studies had a score of three or four (out of five possible); no studies had an affirmative response to the “adverse events” prompt.

### Is There a Relationship Between Dementia Exposure and SCD?

In the current review, regarding the association between dementia exposure and SCD, six studies (18.8%) found significant associations (Studies 1–6; Table 1), 17 studies (53.1%) found no significant association (Studies 7–23; Table 2), and nine studies (28.1%) found mixed associations (Studies 24–32; Supplementary Table 2). Inconsistent associations included differential relationships between exposure and SCD based on the type of measure (i.e., differences between perceptions of decline and frequency of complaints affected by family history [Study 25]; different observed patterns between SCD and SCD-*plus* criteria [Studies 29 and 30]) or usage of the measure (i.e., Study 31 used the *Memory Failures Scale* as a control variable). Unclear associations were primarily found among studies that included participants who either (1) all reported having dementia exposure (Studies 27, 28, and 32) or (2) all reported having memory complaints or SCD (Studies 24 and 26), which reduced opportunities for comparison of the effects.

**Table 2.** Reviewed Articles With Nonsignificant Relationships Between Dementia Exposure and SCD

Reference	Sample type, location	Sample size; age (*SD*)	Study design	Dementia exposure	SCD measures (# items)	Quality appraisal	Analyses and general conclusions
Section 1: Exclusive familial exposure (Articles 7–19)							
7. French (2008)	Community (United States)	*n* = 50; 72.2 (5.4)	Cross-sectional	Family history (# relatives) and emotional closeness	MFQ (64 items)	Good quality	No relationship between MFQ and family history was found (*r* = .17, *p* > .05).
8. Grill et al. (2015)	Clinic, postmortem data (United States)	*n* = 197; Age at death: range <60–100+ years.	Cross-sectional	Family history	Participant-reported decline in memory	Lesser quality	Using χ^2^ analyses^a^, no association was found between family history of dementia and SMC (*p* = .47).
9. Hodgson and Cutler (2003)	Community (United States)	Children: *n* = 108; 50.0 (5.6) Controls: *n* = 150; 49.4 (5.6)	Cross-sectional	First degree	SIME (12-items); How do you rate your memory at the present time? Does your ability to remember cause you any worry?	Good quality	Family history was not associated with memory rating (*r* = .035, *p* > .05) or SIME score (*r =* −0.008, *p* >.05), but was associated with memory worry (*r* = −0.209, *p* < .001).
10. Kessler et al. (2021)	Community, referred to clinic (Germany)	*n* = 958; 72.3 (8.1)	Cross-sectional	First and second degree	Self-perceived worsening or frequent problems (*yes/no*) in domains of cognition (e.g., memory, language, attention, orientation)	Good quality	Family history was not associated with subjective cognitive concerns (*r* = 0.04, *p* = .26).
11. La Rue et al. (1996)	Community (United States)	Family history: *n* = 61; 60.9 (8.5) Controls: *n* = 41; 61.9 (10.7)	Cross-sectional	First degree	MFQ (64-items)	Good quality	Using regression^a^, group membership (control vs total sample AD relatives) did not predict MFQ scores (all *p* > .05). A difference was found between early-onset and control participants in some dimensions of the MFQ (*p* < .05); this was not found in late-onset.
12. Lui et al. (2013)	Clinic, noninpatient (China)	*n* = 162; 61.9 (8.4)	Cross-sectional	First degree	Complaint of memory impairment (*yes/no*)	Good quality	Using χ^2^ analyses^a^, no difference in SMC was found between groups (paternal history, maternal history, and no history; *p* > .05).
13. McPherson et al. (1995)	Community (United States)	Family history: *n* = 25; 59.9 (6.9) Controls: *n* = 26; 60.1 (10.8)	Cross-sectional	First degree	MFQ (64-items)	Lesser quality	Using mean difference comparisons (i.e., ANOVA^a^), MFQ scores did not differ between participants with family history of AD and controls (*p* > .05).
14. Mosconi et al. (2007)	Community (United States)	Maternal: *n* = 16; 63.0 (8.0) Paternal: *n* = 8; 67.0 (8.0) Controls: *n* = 25; 69.0 (8.0)	Cross-sectional	First degree	Presence or absence of subjective memory complaint (*yes/no*)	Good quality	Using χ^2^ analyses^a^, no difference in SMC was found between groups (paternal history, maternal history, and controls; *p* > .05).
15. Nicholas et al. (2017)	Community (United States)	*n* = 1,148–1,261 (differs by analysis); 53.6 (6.6)	Longitudinal	First degree	Frequency of Forgetting (FF) subscale of MFQ (18-items) IQCODE (16-items)	Good quality	Exposure was not associated with FF (*r* = −0.04) or IQCODE (*r* = 0.014) at baseline. Using multilevel analysis, these relationships did not differ over time (*b* = −1.126, *SE* = 0.792; *b* = 0.168, *SE* = 0.294, *p* > .05).
16. Pavisic et al. (2021)	Community (United States)	*n* = 460; 70.7 (0.70)	Cross-sectional	1^st^ Degree	MyCog (24-items) *SCD-plus* items: (1) Do you perceive memory or cognitive difficulties, (2) In the last 2 years has your cognition or memory declined. If yes, follow-up questions were asked (onset-age, peer comparison, help-seeking).	Good quality	Family history was not a significant predictor of MyCog score (*b* = 0.25, 95% CI: −0.67, 1.17, *p* = .595).
17. Reynolds et al. (2022)	Community (United States)	SCD: *n* = 113; 67.4 (range 50–87) Non-SCD: *n* = 253; 67.2 (range 50–88)	Cross-sectional	Family history	Have you experienced a change in your memory in the last 1–3 years? Has this been a persistent change over the last 6 months? Are you concerned about this change?	Good quality	There were no significant differences between groups (SCD, non-SCD) in rates of family history, χ^2^(1) = 2.07, *p* = .151.
18. Rimajova et al. (2008)	Community (Australia)	SMC yes: *n* = 23; 65.0 (9.3) SMC no: *n* = 7; 66.7 (11.3)	Cross-sectional	Family history	“SMC status was determined through responses to the CAMDEX-R relating to subjective memory problems” (p. 139).	Lesser quality	Using ANOVA and χ^2^ analyses^a^, no differences between groups (SMC, no SMC) were found on family history (*p* = .195) or CAMCOG-R (*p* = .06).
19. Small et al. (1994)	Community (United States)	Family history: *n* = 29; 60.6 (9.3) Controls: *n* = 14; 59.2 (11.7)	Longitudinal	First degree	MFQ (64-items)	Good quality	Student *t* tests^a^ found no significant difference between those with and without family history on the MFQ (*p* > .05).
Section 2. Familial and nonfamilial exposure (Articles 20–23)							
20. Heun et al. (2003)	Clinic and community (Germany)	Relatives: AD: *n* = 238; 67.9 (10.2) MD: *n* = 290; 68.5 (10.9) Control: *n* = 190; 64.5 (9.8) Spouses: AD: *n* = 59; 68.9 (9.6) MD: *n* = 74; 65.8 (8.1) Control: *n* = 63; 66.1 (9.1)	Cross-sectional	First degree; Spouse of patient with AD, MD, or control.	“…assessed by questioning the subjects whether they believed that their memory abilities had become worse in comparison with earlier periods of life, and if so, if this had caused problems. When the subjects reported memory complaints and provided a consistent example, the age at onset of memory complaints and of the related problems was assessed.” (p. 79).	Good quality	Using χ^2^ analyses, memory complaint prevalence did not differ among relatives of patients with AD, MD, or control (χ^2^ estimates = 0.017–4.58, *p* = .03–.89). After controlling for age and gender, this result was reduced to nonsignificance. Cox proportional hazards = 0.74–0.85, all 95% CI: <1.0.
21. Jeffers et al. (2021)	Community (United States)	Caregivers with SCD: *n* = 2,670 Caregivers, no SCD: *n* = 18,568, Non-caregivers: *n* = 72,366 Ages 45–64 and ≥65 years.	Cross-sectional	Dementia caregiving	“Respondents were classified as experiencing SCD if they responded affirmatively when asked if they had experienced worsening or more frequent confusion or memory loss in the past 12 months.” (p. 1592)	Good quality	Mean difference comparisons (i.e., *t* tests) and modified χ^2^ analyses^a^ found a significant difference between caregivers and non-caregivers in SCD rates (*p* < .001), but within caregivers there was no difference between type of care provided or if the care recipience had a dementia/AD diagnosis (*p* = .70).
22. Kinzer and Suhr (2016)	Community (United States)	*n* = 100; 69.2 (8.5)	Cross-sectional	First and second degree; Close friend or spouse	Memory Controllability Inventory (19-items)	Good quality	No difference in SMC was found between those with and without dementia exposure, *F*(1, 95) = 1.61, *p* = .21. Dementia worry was correlated with memory concern among those with nongenetic exposure only (*r* = 0.39, *p* = .004), not among genetic (*r* = 0.21, *p* = .37) or no exposure (*r* = 0.26, *p* = .18).
23. Lee et al. (2021)	Community (United States)	*n* = 126; 65.1 (8.7)	Cross-sectional	First and second degree; Close friend or spouse; Frequency of contact.	EMQ-R (13-items)	Good quality	No group (genetic, nongenetic, and no exposure) differences were found in SMC (*F*(2, 123) = 0.41, *p* = .66), or in subjective memory decline (χ^2^(2) = 1.19, *p* = .55).

*Notes*: AD = Alzheimer’s disease; ANCOVA = analysis of covariance; CAMDEX-R = revised cambridge examination for mental disorders of the elderly; EMQ-R = everyday memory questionnaire – revised; IQCODE = informant questionnaire on cognitive decline in elderly; MD = major depression; MFQ = memory functioning questionnaire; SCD = subjective cognitive decline; SIME = short inventory of memory experiences; SMC = subjective memory complaints; Quality appraisal was determined using the LEGEND framework. First degree = parent, child, and sibling; second degree = aunt, uncle, grandparent, niece, and nephew.

^a^Indicates that specific results and estimates (e.g., χ^2^, *t*-test, *F*-value, etc.) were not provided in the text, such that only significance values (*p* values) were presented.

Among the six studies that found a significant association between dementia exposure and SCD, the primary result was that adults who reported a family history of dementia (Studies 1–4) or were spouses of persons with AD (Studies 5–6) typically reported more frequent cognitive problems and had worse self-perceptions of their memory functioning compared with those without a family history. The clearest evidence for a positive relationship between dementia exposure and SCD was found in four studies that specifically contrasted individuals with dementia exposure to those without any family history (Studies 1 and 4–6). For example, in the one study that compared participants with MCI to participants without cognitive impairment (i.e., cognitively normal), when cognitively normal participants had a family history of dementia, they reported similar rates of SCD to those with MCI (Study 4). This suggests that familiarity and experience with dementia may spark comparable levels of self-perceived impairment as those with objective cognitive impairment.

The 17 studies that found no significant relationship between dementia exposure and SCD tended to have small sample sizes (*n*s < 200) or focused on dementia worry as the primary target of the study, with SCD included as a supplemental variable. Although no significant relationships were found between the key variables of interest, there were several commonalities in these studies with null findings. First, four had sample sizes of less than, or equal to, 50 participants (Studies 7, 14, 18, and 19), with an additional six studies with samples of less than 200 participants (*n*s = 51–197; Studies 8, 11–13, 22, and 23). This suggests that the relationship may not have been able to be detected due to power, given estimates of small- or small-to-medium effect sizes within those studies which did find a significant relationship (e.g., odds ratio [OR] = 1.21 [Study 1], *r* = 0.127 [Study 3], and η^2^ = 0.03 [Study 6]). One caveat to this general pattern of small samples was Study 21, which used a large national sample (*n* = 93,604) of middle-aged and older U.S. adults. This study examined the difference between individuals who provide care to others and those that do not and found that caregiving was associated with significantly greater rates of SCD, but that the type of care provided or whether the care recipient had dementia did not affect this relationship. Second, four studies examined subjective cognition as a predictor or modifier of the relationship between dementia exposure and fear or perceived risk of dementia or AD (Studies 7, 9, 22, and 23), however, no significant differences between exposure and SCD were found.

### What Potential Factors Influence the Relationship Between Dementia Exposure and SCD?

To provide context for the relationship between dementia exposure and SCD, as well as potentially clarify nonsignificant and unclear results, four factors were exploratorily identified based on commonalities within the included studies. These factors included: (1) proximity of the social/familial exposure, (2) awareness of potential genetic susceptibility (i.e., APOE status), (3) the role of dementia worry, and (4) sensitivity of the measures.

Most studies (26 of 32 total) used an exclusively familial definition of dementia exposure, such that a positive family history of dementia or AD was the indicator. A majority (57.7%) further specified that dementia exposure was limited to first-degree relatives, with only a subset including second-degree (Studies 10, 22, 23, and 31) or family history without specification of proximity (Studies 2, 7, 8, 17, 18, 24, 26, and 30). Studies 5 and 6 found a positive association between nonfamilial experience with dementia and SCD, such that close or long-term proximity had a similar effect to positive family history. Specifically, caring for or having a spouse with dementia resulted in greater reported subjective cognitive problems and greater perceived AD risk, while simply knowing someone or having a nonfirst-degree relative with dementia or AD was no different than having zero exposure.

Relatedly, another factor that could influence SCD is awareness of one’s status as a carrier of the APOE gene. APOE status was not a central focus of the current review, in favor of differing types of dementia exposure, thus only eight studies included APOE status (Studies 14–16, 18, 24, 27, 29, and 30). Within these studies, participant awareness of APOE status was unclear (i.e., one specified participant were blinded to their status and the other seven did not state it). Amongst six of eight studies, APOE status did not relate to SCD measures, and in one case (Study 30) all participants were categorized as having SCD, and only the “convenience” (compared with “population”-based) sample had biomarker data, which did not allow for the comparison of SCD-*plus* criteria. One study (29) that found a mixed association between SCD and dementia exposure, also found a significant relationship between SCD-*plus* scores and greater biomarker levels (assessed via amyloid), but this relationship was only significant among individuals with a family history of AD.

The third factor that may modify the relationship between dementia exposure and SCD is personal perceptions of dementia, which include dementia worry, perceptions of dementia risk, or attributions of cognitive concerns to dementia. In the current review, seven studies assessed the role of personal perceptions of dementia and dementia worry (Studies 5, 7, 9, 22, 23, 26, and 31). Two consistent features emerged: (1) greater perceived cognitive impairment was associated with greater fear, worry, or attributions to dementia, and (2) exposure to dementia or family history tended to increase perceptions or risk, fear of dementia, or intentions to seek cognitive examinations. Two studies found that subjective memory complaints (Studies 7 and 23) were related to dementia worry and fear of developing AD, while dementia exposure was not similarly associated. Ostergren and colleagues (2017, Study 5) found subjective memory rating was associated with perceived risk of AD among individuals with first degree or spousal exposure, but not among individuals who only knew someone with dementia.

The final consideration that may potentially modify the pattern of results regarding SCD and dementia exposure is the measures used to assess subjective memory or cognition. Almost one third of the studies reviewed (Studies 2, 4, 5, 8, 12, 14, 21, 24, and 32) used a single item to assess or classify individuals as having SCD. Only three studies found a significant relationship between these single-item measures and dementia exposure, suggesting that a single-item may not be sufficient for capturing the range of symptoms related to subjective memory or cognitive differences. To contrast, among studies that used a multi-item scale or multiple types of assessment, 66.7% (10 of 15 studies) found either mixed or significant evidence for the relationship between dementia exposure and SCD.

## Discussion and Implications

Personal experience with dementia, whether through familial or nonfamilial exposure, may be associated with self-perceptions of memory or cognition, yet the relationship between these two variables has not been comprehensively evaluated. In the present review, slightly less than 20% of studies (6 out of 32) found evidence of a positive relationship between dementia exposure and SCD, such that having dementia exposure was associated with poorer perceptions of cognitive and memory functioning. This result is in line with past work that found greater vigilance for potential symptoms of cognitive decline among those with a first-degree relative with AD compared with those without such family history (e.g., Hodgson & Cutler, 2003). In contrast, slightly more than half of the reviewed studies (17 of 32) found no significant relationship between dementia exposure and SCD. Although a majority of reviewed studies found no relationship, studies that did find an association tended to be stronger methodologically, thus other factors (including methods) may have affected the interpretations or lack of findings (reviewed below). Although the relationship between these two variables should be interpreted carefully, when considering the totality of the evidence, including relationships with fear of AD or dementia worry, this review suggests that dementia exposure should be considered and included in future work on SCI and SCD.

### Differential Impacts of Dementia Exposure Types

The current review used an inclusive search of dementia exposure types, which included familial (i.e., first-degree relative, second-degree relative, and general family history) or nonfamilial (i.e., spouse, partner, and close friend) exposures. Although there are arguments for and against such a broad definition of dementia exposure (compared with a narrower definition), by including many types of exposure this review was able to preliminarily investigate the relative impact of family history versus familiarity and emotional closeness. We assert that dementia exposure can encompass many relationships with people with dementia. However, the vast majority of studies used a familial definition of dementia exposure, such that only two studies (i.e., Jeffers et al., 2021 and Vitaliano et al., 2017) did not include a familial definition of dementia exposure. Vitaliano et al. (2017) exclusively used spouses of persons with AD as their sample (another type of familial exposure), while Jeffers et al. (2021) compared caregivers with and without SCD, but did not specify the relationship to the care recipient, thus it cannot be ruled out that the care providers are also relatives. Furthermore, almost half of the studies (15 of 32 reviewed) specified that dementia exposure was categorized as a first-degree family (i.e., parent, sibling, and child) only, which potentially limits the types of conclusions that can be drawn. Specifically, assessing first-degree family history does not necessarily address the frequency of contact, emotional or subjective closeness, or other social circumstances, such as caregiving or spousal relationships.

A prime example of the importance of parsing the different types of dementia exposure (compared with limiting to family history only) that we identified in the current review was the differential patterns of results obtained between individuals who had a positive APOE status (i.e., genetic susceptibility to AD) and those who were caregivers or spouses of those with dementia (i.e., nonfamilial exposure) on SCD reporting. A large body of work (see Verghese et al., 2011 for review) has found support for the role of APOE and its various subtypes as a risk factor for AD and related dementias. Similarly, some studies have examined the interplay between awareness of potential genetic susceptibility to AD and how individuals may interpret perceived cognitive changes (Lineweaver et al., 2014), fear of dementia (Hodgson & Cutler, 2003), or willingness to seek a test for AD (Cutler & Hodgson, 2003). Yet our review found that in most studies that included APOE status, this factor was not related to SCD. In contrast, two of the reviewed studies that included spouses and/or caregivers of persons with dementia found significant relationships between exposure and SCD, such that participants both perceived greater personal risk of AD and reported more cognitive problems. Although these results are preliminary, based on this synthesis the role of APOE status appears to be relatively less impactful compared with frequency of contact or proximity of the relationship with the individual who has dementia, but one large caveat is that participant awareness of APOE status was largely unknown in the current selection of studies.

### Dementia Exposure and Dementia Worry

Another important consideration is that dementia exposure captures a different experience than dementia worry, which in turn may differentially affect SCD, yet these constructs are often examined simultaneously. In the present review, several studies focused on the relationship between dementia exposure and perceived risk of AD or fear of experiencing dementia (i.e., *dementia worry*; Kessler et al., 2012; Kinzer & Suhr, 2016), and included subjective memory or cognition as potential predictors or modifiers of this relationship (e.g., worse self-perceived memory associated with greater AD symptom-seeking; Hodgson & Cutler, 2003). As the relationship between dementia exposure and SCD is mixed, more research should first establish this link before examining dementia exposure as a modifier of other relationships, especially as it is not clear which variable would necessarily be the moderator (i.e., it is conceivable that dementia exposure precedes dementia worry and vice versa). Similarly, the relationship between dementia exposure and dementia worry is unclear. Some studies have found that individuals who have relatives with AD or dementia are more concerned about potential signs and symptoms of cognitive impairment (Hodgson & Cutler, 2003), and may be more likely to attribute memory problems to potential cognitive impairment (Mills et al., 2023). In contrast, other studies have found no relationship between dementia exposure and dementia worry (e.g., French, 2008), while evidence of dementia worry among those without any exposure perhaps suggests a “fear of the unknown” (e.g., Cutler & Hodgson, 2001; Kessler et al., 2012). Given the complexities of these underlying relationships and the robable dynamic associations, we believe there is a need for both more foundational investigation of these variables in isolation as well as in tandem. The examination of dementia exposure and dementia worry is still relatively nascent, with many studies only appearing in the last two decades, thus there are likely to be many important discoveries on the horizon with implications for well-being and cognitive functioning.

### Methodological Considerations for Dementia Exposure and Subjective Cognitive Decline

A key conclusion from the review of this literature is the need for more refined measures of both dementia exposure and SCD. As noted above, dementia exposure is generally assessed through the social or familial proximity of the individual with AD to the reporter. This operationalization lacks information about the extent to which the reporter personally identifies with the target individual, the emotional closeness of that relationship, as well as other social processes that might lead to the incorporation of the dementia exposure into the reporter’s perceptions of their own cognition in meaningful ways (Cavanaugh et al., 1998). Measures that are able to capture the internalization of dementia exposure (or lack thereof) as well as how this process changes over time would provide a clearer understanding of how dementia exposure influences perceptions of one’s own cognitive functioning. Similarly, multiple studies included limited measures of SCD, including single-item measures (e.g., *Do you have problems with your memory?*). The SCD Initiative recommends the use of multi-item measures that assess specific instances of cognitive problems in addition to perceptions of declines in cognitive functioning over time (Rabin et al., 2015) as these measures have better sensitivity and specificity due to the improved reliability and more comprehensive assessment (Rabin et al., 2020). Indeed, in the current review, two studies (i.e., Wolfsgruber et al., 2022; Zhao et al., 2021) illustrated the importance of multi-item measures and broader assessments by demonstrating differential relationships between dementia exposure and general SCD classification compared with the SCD-*plus* criteria. Furthermore, combining more sensitive measures of both dementia exposure and SCD would provide a nuanced understanding of not only whether dementia exposure influences perceptions of cognition but also the mechanisms through which this occurs. For example, an individual with a first-degree relative with AD may only incorporate this into their perceptions of their own cognition if they identify closely with this relative and have frequent contact with them. Even then, the influence may be limited to those functions (e.g., planning and memory for words) that the relative is unable to perform consistently. It is likely a complex process that has not been, as yet, fully assessed.

### Limitations and Future Directions

Given the potential importance of this review for future work in SCI and SCD, it is important to note some limitations to these results. The current review identified several studies that found mixed evidence for the relationship between dementia exposure and SCD, but a main factor that influenced our interpretation of the results within these studies is that they contained participants who either all had cognitive or memory complaints, or all had dementia exposure. Without a control or comparison group (i.e., some participants without a family history, or SCD) it is challenging to draw strong conclusions about the relationship between these key variables. Similarly, our search strategy permitted the inclusion of multiple types of dementia that were not limited to AD, thus many of the results included general reference to experience with dementia. It is possible that by not limiting the search to exclusively AD and related dementias we may have influenced the relationship between our variables of interest, as cognitive complaints related to AD may be distinctly different than those related to a differential diagnosis. For example, someone with exposure to Vascular Dementia may not overly attend to perceived cognitive changes, given that the underlying cause of the dementia is less likely to be inheritable (Alzheimer’s Society, 2022). However, one important note is that many of the studies specified that participants only had experience with a family history of AD (18 of 32 possible studies), which potentially minimizes this limitation. A second limitation that also potentially stems from the current search strategy is the minimal representation of other forms of dementia exposure outside of friends and family. Past work has found support for the role of exposure via work experience in formal caregiving or long-term care settings affecting perceptions of *dementia worry* and anxiety about aging (Kessler et al., 2014), thus it seems probable that these settings would also affect experiences of SCD. The current study was limited to “community-based” samples through our search strategy and inclusion/exclusion criteria; therefore, it is likely that studies investigating this relationship among the formal caregiving workforce were missed. Consequently, formal caregiving exposure remains an important future direction for research. A third limitation is the role of APOE status as a risk factor or modifier of the relationship between SCD and dementia exposure. Some studies have examined the interplay between awareness of potential genetic susceptibility to AD and how individuals may interpret perceived cognitive changes (Lineweaver et al., 2014). In the current study, we cannot draw a strong conclusion to clarify the role of APOE status on SCD, as we only had eight studies that assessed APOE status and it was not a focal interest in the search strategy. Further, of the eight studies reviewed, seven did not state whether participants were aware of their APOE status, while only one study explicitly stated that participants were unaware (i.e., Nicholas et al., 2017); it is probable that awareness of one’s status may affect perceptions and reporting of SCD.

The findings of this review highlight several important areas for future research. First, the inconsistency of results across studies suggests that there is a complexity to the relationships between dementia exposure and SCD that is not adequately understood. As one example, it is necessary to consider other processes that might link dementia exposure and SCD outside of dementia worry. It is possible that social comparison processes such as identification with the individual with dementia, lead the observer to different conclusions about their own cognition. Some individuals may engage in downward social comparison (e.g., “at least my memory isn’t that bad”) rather than in greater worry. This and other factors identified in this review may influence the relationship (exposure proximity, awareness of APOE status, dementia worry, and sensitivity of measures) and should inform future studies examining moderators of these relationships or differential effects across subgroups. Second, none of the studies we identified used qualitative or mixed methods. This is an important area for future work, as exploring the meaning attributed to dementia exposure and *how* it may influence one’s perceptions of cognition as they age is critical to understanding relationships and opportunities for intervention. Although this review identified a body of evidence examining these relationships, exploratory research on the psychosocial mechanisms underlying links between dementia exposure and subjective cognition would develop a deeper understanding.

## Conclusion

This review comprehensively evaluated the current evidence of the association between dementia exposure and SCD and found mixed support for a relationship between these variables that was more heavily weighted toward the absence of an association, yet several factors were also identified that seem equally important to consider. Specifically, the consistency obtained between the roles of worry or fear of AD, family history, and vigilance toward perceived changes in memory or cognition, suggests that these components should be more fully explored in future studies, especially those that use longitudinal methods in order to better capture the temporality of these relationships. Overall, while a consistent relationship was not clearly established between dementia exposure and SCD, we believe that the potential impact of dementia exposure should not be minimized in future work, and in fact should be broadened to include other important considerations such as frequency of contact, emotional closeness, and other forms of nonfamilial dementia experience to better identify individuals who may be potentially at risk of future subjective or objective cognitive decline.

## Supplementary Material

igad056_suppl_Supplementary_Material

## Data Availability

This systematic review was registered on PROSPERO (ID: CRD42021232329) prior to conducting the formal literature search; a review protocol was not prepared. The link to this preregistration can be found at: https://www.crd.york.ac.uk/prospero/display_record.php?RecordID=232329. In accordance with the promotion of transparency, openness, and reproducibility, materials and data supporting this synthesis are available upon request.
