# Supplementary material for: How Does Exposure to Dementia Relate to Subjective Cognition? A Systematic Review
Source: Innov Aging. 2023 Jun 19;7(6):igad056. doi: 10.1093/geroni/igad056 (PMC10368315; doi:10.1093/geroni/igad056)
Supplement: igad056_suppl_Supplementary_Material [file igad056_suppl_supplementary_material.docx]

**Supplementary Table 1.** *Adapted LEGEND Evidence Appraisal Guidelines*

| **Category** | **Good Quality** | **Lesser Quality** | |
| --- | --- | --- | --- |
| **Overview** |  |  | |
| - Do the study aim/purpose/objectives assist in answering your research question? | | Yes (1) | No (0) or Unknown (0) |
| - Do the inclusion/exclusion criteria assist in answering your research question? | | Yes (1) | No (0) or Unknown (0) |
| - Is the study design congruent with the author’s study aim/purpose/objectives above? | | Yes (1) | No (0) or Unknown (0) |
| **Validity: Are the Results of the Study Valid or Credible?** |  |  | |
| - Were the study methods appropriate for the question? | Yes (1) | No (0) or Unknown (0) | |
| - Were instruments used to measure the outcomes valid and reliable? | Yes (1) | No (0) or Unknown (0) | |
| - Were all appropriate variables and interventions clearly described? | Yes (1) | No (0) or Unknown (0) | |
| - Were all appropriate outcomes clearly described? | Yes (1) | No (0) or Unknown (0) | |
| - Were all participants accounted for at the conclusion of the study? | Yes (1) | No (0) or Unknown (0) | |
| - Was there freedom from conflict of interest? | Yes (1) | No (0) or Unknown (0) | |
| **Reliability: Are These Valid Study Results Important?** |  |  | |
| - Were the statistical analysis methods appropriate? | Yes (1) | No (0) or Unknown (0) | |
| - Did the study have a sufficiently large sample size? | Yes (1) | No (0) or Unknown (0) | |
| - What are the main results of the study? | Yes (1) | No (0) or Unknown (0) | |
| - Were the results statistically significant? | Yes (1) | No (0) or Unknown (0) | |
| - Were the results clinically significant? | Yes (1) | No (0) or Unknown (0) | |
| - Were adverse events assessed? | Yes (1) | No (0) or Unknown (0) | |
| **Evaluating the Quality Level/Evidence Level**   - Consider each “*No*” answer and the degree to which this limitation is a threat to the validity of the results, then check the appropriate box to assign the level of quality for this study/article. - Consider an “*Unknown*” answer to one or more questions as a similar limitation to answering “*No,*” if the information is not available in the article. | Total score greater than or equal to 9 points (out of 15 maximum). | Total score less than or equal to 8 points (out of 15 maximum). | |

*Note*. This table has been adapted from the Cincinnati Children’s Hospital Medical Center and CCHMC Evidence Collaboration (<https://www.cincinnatichildrens.org/evidence>). The original version referred to “clinical questions” of interest (herein edited to “research questions of interest”) and included applicability ratings to aid in clinical decision-making.

**Supplementary Table 2.** *Reviewed Articles with Mixed Association Between Dementia Exposure and SCD*

| **Reference** | **Sample Type, Location** | **Sample Size; Age (*SD*)** | **Study Design** | **Dementia Exposure** | **SCD Measures (# items)** | **Quality Appraisal** | **Analyses and General Conclusions** |
| --- | --- | --- | --- | --- | --- | --- | --- |
| *Section 1. Exclusive Familial Exposure (Articles 24-30)* | | | | | | | |
| Abdelnour et al. (2017), #24 | Clinic and Community (Spain) | MU: *n* = 106; 64.5 (9.8)  OHI: *n =* 220; 63.4 (7.8) | Cross-sectional | Family history | Do you feel like your memory is worsening?  Do you worry about your memory problems? | Good Quality | All participants were classified as having SCD and were worried about their memory. The OHI sample had higher rates (62.7%) of family history than the MU sample (41.5%; χ^2^ = 13.06, *p* < .001). |
| Mogle et al. (2020), #25 | Community (U.S.) | *n* = 454; 76.6 (4.8) | Longitudinal | 1^st^ Degree | In the past year, how often did you have trouble remembering things?  Compared with one year ago, do you have trouble remembering things more often, less often, or about the same?  Compared with 10-years ago, do you have trouble remembering things more often, less often, or about the same? | Good Quality | Frequency of memory problems was marginally related to family history at baseline (*b* = .11, *SE* = .06, *p* = .06), but not over time. Family history was associated with perceived 1-year decline (*OR* = 2.41, 95% CIs 1.36, 4.25) at baseline, but not over time. Family history was not related to 10-year decline (*p*s > .49). |
| Ramakers et al. (2009), #26 | Outpatient clinic and Community (Netherlands) | Memory Clinic: *n* = 33; 62.0 (8.6)  Controls: *n* = 83; 65.1 (10.3) | Cross-sectional | Family history | Do you find yourself forgetful? (*yes/no*)  MIA (34-items)  DECO (19-items) | Lesser Quality | All participants reported memory complaints. Using *t*-tests and χ^2^ analysesª, clinic participants performed worse than control on both the MIA-MSE (*p* < .01) and DECO (*p* = .04). Family history was more common among clinic participants compared to control (*p* = .048). |
| Verfaillie et al. (2018), #27 | Community (Canada) | SCD: *n* = 68; 64.0 (5.0)  No SCD: *n* = 56; 64.0 (5.0) | Longitudinal | 1^st^ Degree | Do you think your memory is becoming worse? (*yes/no*)  E-Cog (39-items) | Good Quality | All participants had family history of AD. Using *t*-tests and χ^2^ analysesª, there were no significant differences between groups (SCD, no SCD) on parent (*p* = .24) or sibling (*p* = .39) exposure. There were significant group differences on ECOG were found on the dimensions of memory, language, and attention (all *p*s < .01). |
| Werner & Heinik (2004), #28 | Clinic (Israel) | *n* = 93; 50.7 (8.1) | Cross-sectional | 1^st^ Degree | CAMCOG (3-items): “Participants were asked to report whether they suffered problems with remembering things or names of close relatives, or with their orientation in place and time” (p. 480). | Good Quality | All participants had family history of dementia (48.8% were caregivers) and memory functioning was high (*M* = 1.5, *SD* = 0.4; range 1-4). Participants with more subjective memory problems endorsed greater intention of seeking a cognitive assessment across models (*β*s = .30-.37, all *p*s < .05). |
| Wolfsgruber et al. (2022), #29 | Community (Germany) | AD Relatives: *n* = 82; 65.6 (4.5)  Control: *n* = 236; 68.9 (5.4) | Longitudinal | 1^st^ Degree | Subjective Cognitive Decline Interview (SCD-I) for 5 domains (memory, language, planning, attention, other).  SCD-*plus* questions: presence of worries or onset of decline for each domain. | Good Quality | No significant group (AD relatives, control) differences in SCD were found (*p* > .05). There were significant differences in SCD+ scoring: χ^2^(1) ranges = 4.36-17.2, *p*s = .04 to < .001). Higher SCD+ scores at baseline predicted worse cognitive trajectories in AD relatives (*b* = -.077, *SE* = .034, *p* = .024), but not controls (*b* = -.024, *SE* = .023, *p* =. 299). |
| Zhao et al. (2021) #30 | Community (China) | Convenience: *n* = 212; 65 (range 63-69)  Population: *n* = 110; 67 (range 63-70) | Cross-sectional | Family history | Semi-structured SCD interview for 5 domains (memory, language, planning, attention, other): "Do you think your memory has become worse?"  If yes, follow-up questions about SCD-*plus* features (i.e., worry, onset, peer comparison, help-seeking). | Good Quality | All participants were classified as having SCD. The convenience sample had higher rates of family history compared to the population sample (χ^2^ = 22.23, *p* < .001). Using SCD-*plus* criteria, the convenience sample reported more memory worry (χ^2^ = 46.39, *p* < .001), cognitive decline (χ^2^ = 11.84, *p* = .001), and help-seeking (χ^2^ = 6.38, *p* = .012). The population sample reported more difficulties with planning (χ^2^ = 9.99, *p* = .002) and language (χ^2^ = 7.75, *p* = .005). |
| *Section 2. Familial and Non-Familial Exposure (Articles 31 and 32)* | | | | | | | |
| Mills et al. (2020), #31 | Community (U.S.) | *n* = 498; 52.3 (7.6) | Cross-sectional | 1^st^ and 2^nd^ Degree; Other relative; Close friend or spouse | MFS (12-items)  What do you think is the most likely cause of the following memory failures [from MFS]? *(1 = AD/dementia; 0 = all other attributions)* | Good Quality | MFS results were not directly reported, but 490/498 participants reported at least one memory failure on the MFS. Almost half (47.4%) of participants had AD experience, and exposure was associated with memory failure attribution (*r* = .19, *p* < .01). |
| Tsai et al. (2006), #32 | Clinic and community (U.S., Germany, Canada) | SMC yes: *n* = 134; 69.6 (10.5)  SMC no: *n* = 1,365; 68.4 (10.1) | Cross-sectional | 1^st^ Degree; Spouse | Do you have trouble remembering things from one second to the next? | Good Quality | All participants had dementia exposure. Using logistic regression, first-degree relatives were more likely to report SMC than spouses (*OR* = 1.9, 95% CIs = 1.3, 3.0). |

*Notes.* SCD = Subjective Cognitive Decline; Quality appraisal was determined using the LEGEND framework; ª Indicates that specific results and estimates (e.g., χ^2^, *t*-test, F-value, etc.) were not provided in the text, such that only significance values (*p*-values) were presented. MU = Memory Unit; OHI = Open House Initiative; 1^st^ Degree = parent, child, sibling; 2^nd^ Degree = aunt, uncle, grandparent, niece, nephew; U.S. = United States; MIA = Metamemory in Adulthood questionnaire; DECO = Détérioration Cognitive Observée; E-Cog = Everyday Cognition Scale; AD = Alzheimer’s disease; CAMCOG = Cambridge Cognition Examination; MFS = Memory Failures Scale; SMC = Subjective Memory Complaint.

*PRISMA Checklist for Dementia Exposure and Subjective Cognition Systematic Review*


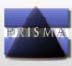
**PRISMA 2020 Checklist**

| **Section and Topic** | **Item #** | **Checklist item** | **Location where item is reported** |
| --- | --- | --- | --- |
| **TITLE** | | |  |
| Title | 1 | Identify the report as a systematic review. | p. 1 |
| **ABSTRACT** | | |  |
| Abstract | 2 | See the PRISMA 2020 for Abstracts checklist. | p. 2 |
| **INTRODUCTION** | | |  |
| Rationale | 3 | Describe the rationale for the review in the context of existing knowledge. | pp. 3-5 |
| Objectives | 4 | Provide an explicit statement of the objective(s) or question(s) the review addresses. | p. 5, lines 17-23 |
| **METHODS** | | |  |
| Eligibility criteria | 5 | Specify the inclusion and exclusion criteria for the review and how studies were grouped for the syntheses. | pp. 6-7, “selection criteria” |
| Information sources | 6 | Specify all databases, registers, websites, organisations, reference lists and other sources searched or consulted to identify studies. Specify the date when each source was last searched or consulted. | p. 6, “search strategy” |
| Search strategy | 7 | Present the full search strategies for all databases, registers and websites, including any filters and limits used. | p. 6, “search strategy” |
| Selection process | 8 | Specify the methods used to decide whether a study met the inclusion criteria of the review, including how many reviewers screened each record and each report retrieved, whether they worked independently, and if applicable, details of automation tools used in the process. | p. 7, “selection and data extraction” |
| Data collection process | 9 | Specify the methods used to collect data from reports, including how many reviewers collected data from each report, whether they worked independently, any processes for obtaining or confirming data from study investigators, and if applicable, details of automation tools used in the process. | p. 7, “selection and data extraction” |
| Data items | 10a | List and define all outcomes for which data were sought. Specify whether all results that were compatible with each outcome domain in each study were sought (e.g. all measures, time points, analyses), and if not, the methods used to decide which results to collect. | p. 7, “selection and data extraction” |
|  | 10b | List and define all other variables for which data were sought (e.g. participant and intervention characteristics, funding sources). Describe any assumptions made about any missing or unclear information. | p. 7, “selection and data extraction” |
| Study risk of bias assessment | 11 | Specify the methods used to assess risk of bias in the included studies, including details of the tool(s) used, how many reviewers assessed each study and whether they worked independently, and if applicable, details of automation tools used in the process. | pp. 7-8, “quality appraisal” |
| Effect measures | 12 | Specify for each outcome the effect measure(s) (e.g. risk ratio, mean difference) used in the synthesis or presentation of results. | p. 7, “selection and data extraction”; Tables 1, 2, and Supplementary Table 2—“analyses and general conclusions” column |
| Synthesis methods | 13a | Describe the processes used to decide which studies were eligible for each synthesis (e.g. tabulating the study intervention characteristics and comparing against the planned groups for each synthesis (item #5)). | pp. 8-9, “narrative synthesis” |
|  | 13b | Describe any methods required to prepare the data for presentation or synthesis, such as handling of missing summary statistics, or data conversions. | N/A |
|  | 13c | Describe any methods used to tabulate or visually display results of individual studies and syntheses. | pp. 8-9, “narrative synthesis” |
|  | 13d | Describe any methods used to synthesize results and provide a rationale for the choice(s). If meta-analysis was performed, describe the model(s), method(s) to identify the presence and extent of statistical heterogeneity, and software package(s) used. | pp. 8-9, “narrative synthesis” |
|  | 13e | Describe any methods used to explore possible causes of heterogeneity among study results (e.g. subgroup analysis, meta-regression). | pp. 8-9, “narrative synthesis” |
|  | 13f | Describe any sensitivity analyses conducted to assess robustness of the synthesized results. | N/A |
| Reporting bias assessment | 14 | Describe any methods used to assess risk of bias due to missing results in a synthesis (arising from reporting biases). | pp. 7-8, “quality appraisal”; Supplementary Table 1 |
| Certainty assessment | 15 | Describe any methods used to assess certainty (or confidence) in the body of evidence for an outcome. | pp. 7-8, “quality appraisal”; Supplementary Table 1 |
| **RESULTS** | | |  |
| Study selection | 16a | Describe the results of the search and selection process, from the number of records identified in the search to the number of studies included in the review, ideally using a flow diagram. | pp. 9-10, “overview of reviewed studies”; Figure 2 |
|  | 16b | Cite studies that might appear to meet the inclusion criteria, but which were excluded, and explain why they were excluded. | Figure 2 |
| Study characteristics | 17 | Cite each included study and present its characteristics. | Tables 1, 2, and Supplementary Table 2 |
| Risk of bias in studies | 18 | Present assessments of risk of bias for each included study. | p. 10, “results of quality appraisal”; Tables,1, 2, and Supplementary Table 2 |
| Results of individual studies | 19 | For all outcomes, present, for each study: (a) summary statistics for each group (where appropriate) and (b) an effect estimate and its precision (e.g. confidence/credible interval), ideally using structured tables or plots. | Tables 1, 2, and Supplementary Table 2 |
| Results of syntheses | 20a | For each synthesis, briefly summarise the characteristics and risk of bias among contributing studies. | p. 10, “results of quality appraisal”; Tables,1, 2, and Supplementary Table 2 |
|  | 20b | Present results of all statistical syntheses conducted. If meta-analysis was done, present for each the summary estimate and its precision (e.g. confidence/credible interval) and measures of statistical heterogeneity. If comparing groups, describe the direction of the effect. | N/A |
|  | 20c | Present results of all investigations of possible causes of heterogeneity among study results. | pp. 12-14, “what potential factors impact the relationship between dementia exposure and self-reported cognition?” |
|  | 20d | Present results of all sensitivity analyses conducted to assess the robustness of the synthesized results. | N/A |
| Reporting biases | 21 | Present assessments of risk of bias due to missing results (arising from reporting biases) for each synthesis assessed. | N/A |
| Certainty of evidence | 22 | Present assessments of certainty (or confidence) in the body of evidence for each outcome assessed. | pp. 10-12, “is there a relationship between dementia exposure and self-reported cognition?”; Tables 1, 2, Supplementary Table 2 |
| **DISCUSSION** | | |  |
| Discussion | 23a | Provide a general interpretation of the results in the context of other evidence. | pp. 15-19 |
|  | 23b | Discuss any limitations of the evidence included in the review. | pp. 19-21, “limitations and future directions” |
|  | 23c | Discuss any limitations of the review processes used. | pp. 19-21, “limitations and future directions” |
|  | 23d | Discuss implications of the results for practice, policy, and future research. | p. 3, “Translational Significance”, pp. 21-22 |
| **OTHER INFORMATION** | | |  |
| Registration and protocol | 24a | Provide registration information for the review, including register name and registration number, or state that the review was not registered. | p. 6, “methods”; p. 32, “acknowledgements” |
|  | 24b | Indicate where the review protocol can be accessed, or state that a protocol was not prepared. | p. 32, “acknowledgements” |
|  | 24c | Describe and explain any amendments to information provided at registration or in the protocol. | N/A |
| Support | 25 | Describe sources of financial or non-financial support for the review, and the role of the funders or sponsors in the review. | p. 32, “funding” |
| Competing interests | 26 | Declare any competing interests of review authors. | p. 32, “conflict of interest” |
| Availability of data, code and other materials | 27 | Report which of the following are publicly available and where they can be found: template data collection forms; data extracted from included studies; data used for all analyses; analytic code; any other materials used in the review. | N/A |

*From:*  p. MJ, McKenzie JE, Bossuyt PM, Boutron I, Hoffmann TC, Mulrow CD, et al. The PRISMA 2020 statement: an updated guideline for reporting systematic reviews. BMJ 2021;372:n71. doi: 10.1136/bmj.n71

For more information, visit: <http://www.prisma-statement.org/>
